# Predicting Clinically Significant Portal Hypertension in MASLD Using Routine Ultrasound and Clinical Data: A Retrospective Cohort Study

**DOI:** 10.1155/bmri/1662756

**Published:** 2026-04-11

**Authors:** Nebyu Yonas Shanka, Chavdar S. Pavlov, Taisiia A. Turankova, Temesgen Tesfaye Ajabo, Leul Mekonnen Nigatu, Biniyam Esayas Dana

**Affiliations:** ^1^ Department of Therapy, Sechenov First Moscow State Medical University (Sechenov University), Moscow, Russia, mma.ru; ^2^ Department of Internal Medicine, Wolaita Sodo University Comprehensive Specialised Hospital, Wolaita Sodo, Ethiopia; ^3^ Department of Hepatology and Gastroenterology, Botkin Moscow City Clinical Hospital, Moscow, Russia; ^4^ Department of Public Health, Sechenov First Moscow State Medical University (Sechenov University), Moscow, Russia, mma.ru; ^5^ Washington University in St. Louis, St. Louis, Missouri, USA, wustl.edu

**Keywords:** abdominal ultrasound, clinically significant portal hypertension (CSPH), hepatic venous pressure gradient (HVPG), liver stiffness, machine learning, metabolic dysfunction–associated steatotic liver disease (MASLD), noninvasive risk stratification, platelet count, portal vein diameter, risk prediction

## Abstract

**Background:**

Clinically significant portal hypertension (CSPH) is a major determinant of adverse outcomes in metabolic dysfunction–associated steatotic liver disease (MASLD), but reliable noninvasive identification remains challenging. We evaluated whether routinely available ultrasound and clinical variables could help identify CSPH in adults with MASLD.

**Methods:**

We performed a retrospective cohort study of adults with MASLD who underwent HVPG measurement at a tertiary referral centre. Routinely available demographic, laboratory, routine ultrasound and liver stiffness variables were evaluated using logistic regression and internally validated machine learning models to identify CSPH, defined as HVPG ≥ 10 mmHg.

**Results:**

Among 218 adults with MASLD, 44 (20.2%) had CSPH. Compared with patients without CSPH, those with CSPH showed less favourable noninvasive profiles, particularly in ultrasound‐ and stiffness‐based measures (liver stiffness: 9.2 vs. 7.8 kPa; spleen length: 119.3 vs. 115.0 mm), whereas most routine biochemical markers were broadly similar between groups. Overall predictive performance was modest across all evaluated models; random forest achieved the highest discrimination (AUC = 0.67), followed by SVM (AUC = 0.65) and logistic regression (AUC = 0.604). In multivariable analysis, portal vein calibre (OR = 1.60) and liver stiffness (OR = 1.134) showed the strongest independent associations with CSPH, although overall predictive accuracy remained limited.

**Conclusions:**

In adults with MASLD, routinely available ultrasound and clinical variables showed only modest ability to identify CSPH. Although some noninvasive markers were independently associated with CSPH, model performance was insufficient for stand‐alone clinical triage. These findings support further refinement of MASLD‐specific noninvasive prediction strategies rather than immediate clinical implementation.

## 1. Introduction

Metabolic dysfunction–associated steatotic liver disease (MASLD), formerly termed nonalcoholic fatty liver disease (NAFLD), is now the most common chronic liver disease worldwide and an increasingly important cause of liver‐related morbidity and mortality [1–3]. Global estimates suggest that steatotic liver disease affects approximately one quarter of the adult population, with substantial regional variation driven by obesity, Type 2 diabetes mellitus and other cardiometabolic risk factors [1, 2]. In addition to progressive liver injury, MASLD is associated with major extrahepatic complications, including cardiovascular disease, chronic kidney disease and malignancy, which further increase its clinical and economic burden. Consequently, MASLD has become a major public health challenge and an increasingly important target for risk stratification in routine hepatology practice [1–3].

Reflecting its metabolic basis, international expert groups have introduced updated nomenclature and diagnostic criteria that emphasise cardiometabolic dysfunction and distinguish MASLD from alcohol‐related liver disease and other secondary causes of hepatic steatosis [4, 5]. Progression to advanced fibrosis and cirrhosis is the principal driver of liver‐related complications in MASLD, including hepatic decompensation, hepatocellular carcinoma and liver‐related death [6, 7]. Accordingly, current guidance has prioritised the early identification of patients at greatest risk of advanced liver disease and has emphasised the use of noninvasive approaches for risk stratification in clinical practice [8, 9].

Type 2 diabetes mellitus is both a major risk factor for MASLD and a frequent manifestation of the same adverse metabolic milieu. More broadly, MASLD and cardiovascular disease have a close and biologically interconnected relationship, supported by shared pathophysiological mechanisms including insulin resistance, chronic low‐grade inflammation, adipose tissue dysfunction, visceral adiposity, endothelial dysfunction and progressive fibrogenesis [10]. This systemic cardiometabolic framework helps explain why MASLD is associated not only with worsening liver disease but also with increased risks of coronary artery disease, atrial fibrillation, heart failure and all‐cause as well as cardiovascular mortality [11–13]. In parallel, cardiovascular and metabolic dysfunction may further aggravate hepatic injury through haemodynamic, inflammatory and metabolic pathways, reinforcing the bidirectional relationship between liver disease progression and extrahepatic cardiometabolic risk [11–13]. These observations underscore that MASLD should be regarded not only as a liver disease but also as a systemic cardiometabolic disorder.

This broader perspective is particularly relevant to the increasingly recognised bidirectional relationship between MASLD and cardiovascular disease, because the same mechanisms that promote systemic vascular injury and adverse cardiac outcomes may also contribute to intrahepatic vascular dysfunction and fibrogenesis [11–13]. In advanced chronic liver disease, clinically significant portal hypertension (CSPH), conventionally defined as a hepatic venous pressure gradient (HVPG) of at least 10 mmHg, marks a critical haemodynamic threshold associated with the development of varices, hepatic decompensation and worse clinical outcomes [14–17]. HVPG remains the reference standard for the assessment of portal pressure, but it is invasive, resource‐intensive and not universally available [16, 17]. These limitations have driven a strong interest in noninvasive tests that can identify patients with CSPH more efficiently.

In MASLD, noninvasive assessment of portal hypertension is particularly challenging because obesity, steatosis and body habitus may reduce the performance or availability of some standard noninvasive approaches [14, 15]. Liver stiffness measurement has emerged as one of the most useful noninvasive markers of advanced fibrosis and portal hypertension [18], but its performance may be affected by technical limitations, and it does not directly capture all of the structural and haemodynamic consequences of portal hypertension [15, 19]. Portal hypertension also produces extrahepatic changes that may be detectable using routine imaging and laboratory tests, including splenic enlargement, increased portal vein calibre and thrombocytopenia related in part to portal congestion and splenic sequestration [19–21].

A substantial body of literature has examined spleen stiffness as a noninvasive predictor of CSPH and oesophageal varices across different chronic liver disease populations [20, 22, 23]. However, spleen stiffness assessment usually requires dedicated elastography platforms and specific technical expertise that may not be universally available. By contrast, standard B‐mode ultrasound measurements such as spleen length, spleen diameter and portal vein calibre are widely accessible [18] and can be obtained during routine abdominal imaging [24, 25]. If these simple morphometric features can be combined with routinely available laboratory markers to identify CSPH in MASLD, they may offer a practical and scalable approach to risk stratification, particularly in settings where more specialised testing is unavailable.

Evidence specific to MASLD remains comparatively limited. A recent systematic review highlighted important gaps in the evidence base for the noninvasive assessment of CSPH in MASLD, particularly with regard to the contribution of routine ultrasound findings and simple laboratory markers [14]. Most existing models have been developed in mixed‐aetiology cohorts, and only a minority have been evaluated in MASLD‐predominant populations [14, 15]. At the same time, machine learning methods offer a flexible framework for modelling complex relationships among clinical, imaging and laboratory variables in chronic liver disease, including the prediction of portal hypertension and related complications. Although studies using radiomics and advanced imaging features have reported promising results, these approaches are not yet widely accessible and often depend on complex acquisition and analytical workflows. It therefore remains uncertain whether machine learning methods meaningfully outperform simpler multivariable models when only routine, clinically available inputs are used [24, 26].

In this retrospective cohort study from a tertiary referral centre, we used invasive HVPG as the reference standard to examine whether routinely collected ultrasound morphometric parameters, liver stiffness measurements, body mass index and standard laboratory tests are associated with CSPH in adults with MASLD. We compared multivariable logistic regression with tree‐based and kernel‐based machine learning approaches, with particular emphasis on model discrimination, calibration and the relative contribution of ultrasound and nonultrasound predictors. Our aim was to evaluate the performance and limitations of practical, interpretable, noninvasive models for CSPH based on routinely available data rather than to infer causal relationships or propose a definitive clinical tool. By providing MASLD‐specific data from a well‐characterised cohort undergoing invasive haemodynamic assessment, this study is aimed at informing the future development and external validation of noninvasive risk stratification strategies for MASLD‐related portal hypertension [27, 28].

## 2. Methods

### 2.1. Study Design and Setting

This retrospective cohort study was conducted at Botkin Moscow City Clinical Hospital, a large tertiary referral centre in Moscow, Russian Federation, with specialised hepatology, gastroenterology and portal hypertension services. The study evaluated adult patients with MASLD who underwent HVPG measurement as part of routine clinical care or approved research activity. Clinical, laboratory, procedural, elastography and imaging data were retrieved from institutional clinical databases, electronic medical records, procedural documentation and imaging archives. The study was designed to assess whether routinely available ultrasound and clinical data could predict CSPH in patients with MASLD.

### 2.2. Study Population

Adults aged 18 years or older were eligible for inclusion if they had MASLD and a valid HVPG measurement. MASLD was defined according to contemporary international criteria, requiring evidence of hepatic steatosis in the presence of at least one cardiometabolic risk factor and the absence of another dominant cause of chronic liver disease [1]. Patients were excluded if they had significant alcohol intake, viral hepatitis, autoimmune liver disease, cholestatic liver disease, Wilson disease, haemochromatosis, drug‐induced liver injury or other specific chronic liver disease aetiologies. When more than one HVPG assessment had been performed on the same individual, only the first eligible assessment was retained in order to preserve independence of observations.

To ensure temporal comparability between invasive and noninvasive measurements, the analytic cohort was restricted to patients who had the required clinical, laboratory and ultrasound data obtained within 2 weeks before or after HVPG measurement. Ultrasound examinations were required to meet predefined quality standards for inclusion in the analysis. Examinations with inadequate image quality or incomplete key measurements were excluded. In addition, patients with missing data required for CSPH ascertainment were excluded from the primary analytic cohort.

From an initial pool of 1900 records screened, 1682 were excluded during cohort assembly. The reasons for exclusion were duplicate records (*n* = 186), non‐MASLD diagnoses (*n* = 742), other chronic liver diseases (*n* = 301), inadequate ultrasound quality (*n* = 233) and missing data required for CSPH ascertainment (*n* = 220). The final analytic cohort therefore comprised 218 patients.

**Figure 1:**
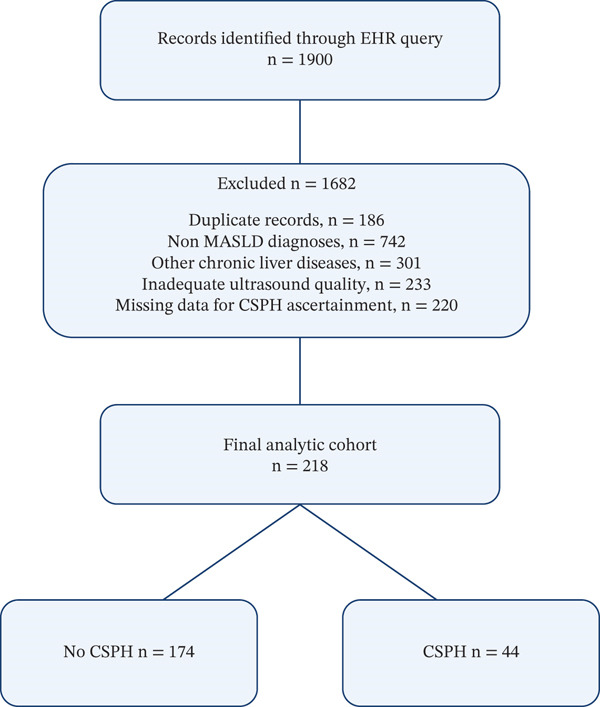
Study flow diagram showing cohort assembly, eligibility and the final analytic sample included in the CSPH analysis. Of 1900 records identified through an electronic health record query, 1682 were excluded because of duplicate records, non‐MASLD diagnoses, other chronic liver diseases, inadequate ultrasound image quality or missing data required to ascertain clinically significant portal hypertension status. A total of 218 patients remained in the final analytic cohort, including 174 without CSPH and 44 with CSPH.

The study selection process is summarised in Figure 1.

### 2.3. Ultrasound and Noninvasive Assessments

Ultrasound examinations were performed by trained radiologists or hepatology sonographers using a standard institutional imaging protocol. Routinely recorded ultrasound variables considered for analysis included portal vein calibre, spleen length and the presence of splenomegaly, ascites or varices, where documented in the clinical imaging record. Liver stiffness measurement, where available as part of routine noninvasive assessment, was also extracted for analysis. The study focused on variables that are commonly obtainable in routine clinical practice and could therefore be realistically incorporated into a noninvasive prediction framework.

Clinical and laboratory variables collected within the same 2‐week time window included age, sex, body mass index, platelet count, serum albumin, international normalised ratio (INR), aspartate aminotransferase, alanine aminotransferase and derived fibrosis indices, including fibrosis‐4 and the AST‐to‐platelet ratio index. Variable selection was guided by clinical relevance, biological plausibility, prior literature on portal hypertension and chronic liver disease severity and practical availability in routine care settings [2, 3].

### 2.4. Reference Standard and Outcome Definition

The primary outcome was CSPH, defined as HVPG of 10 mmHg or greater. HVPG was used as the reference standard because it remains the accepted invasive benchmark for haemodynamic assessment of portal hypertension and risk stratification in chronic liver disease [4, 5]. Patients were classified dichotomously as having CSPH or not having CSPH on the basis of this threshold.

### 2.5. Data Handling and Cohort Assembly

Data were reviewed and harmonised before analysis to ensure consistency across source systems. Where repeated records or overlapping assessments were identified, only the earliest eligible HVPG‐linked observation was retained. Variables were matched to the HVPG date using the predefined 2‐week window. No imputation was performed. Analyses were based on complete cases for the variables included in each model. This approach was chosen to avoid introducing model‐dependent assumptions into a relatively modest‐sized retrospective dataset and to preserve transparency in the relationship between observed predictors and outcomes.

### 2.6. Statistical Analysis

Continuous variables were summarised as medians with interquartile ranges (IQRs), and categorical variables were summarised as counts with percentages. Between‐group comparisons according to CSPH status were performed using the Mann–Whitney *U* test for continuous variables and the Fisher exact test for categorical variables, as appropriate.

The primary inferential analysis used logistic regression to examine associations between candidate predictors and CSPH. Univariable logistic regression models were first fitted for clinically relevant noninvasive variables. Candidate variables for multivariable modelling were selected on the basis of clinical plausibility, availability in routine care and avoidance of unnecessary redundancy among closely related predictors. Adjusted odds ratios (ORs) with 95% confidence intervals (CIs) were reported for the multivariable logistic regression model to support clinical interpretability.

In parallel, machine learning models were developed to evaluate predictive performance using the same routinely available predictor set. The evaluated approaches included logistic regression, random forest and support vector machine. Model training and internal evaluation were performed using five‐fold stratified cross‐validation in order to preserve the class distribution of CSPH across folds and to provide internally validated estimates of performance [6]. For support vector machine models, predictor scaling was applied where appropriate to support stable model fitting. For tree‐based models, feature importance measures were used to summarise the relative contribution of candidate predictors.

Model discrimination was assessed using the area under the receiver operating characteristic curve (AUROC). Calibration was assessed using Brier scores and calibration plots, in accordance with current recommendations for prediction model reporting and appraisal [6, 7]. In addition to global performance metrics, threshold‐based measures including sensitivity, specificity, positive predictive value and negative predictive value were examined at selected probability thresholds to provide clinically interpretable information regarding classification behaviour.

### 2.7. Methodological Considerations

Because this was a retrospective study based on a selected tertiary‐care cohort, the findings are intended primarily to support model development and internal performance assessment rather than causal inference. The use of HVPG as the reference standard strengthens the validity of outcome classification; however, the requirement for invasive haemodynamic testing together with temporally matched noninvasive data may limit generalisability to broader MASLD populations encountered in general practice. In addition, some candidate predictors, particularly ultrasound‐derived measurements, remain operator‐dependent despite the use of a standard institutional protocol, and intercentre variation should be considered when interpreting external applicability.

## 3. Results

### 3.1. Study Population and Prevalence of CSPH

A total of 218 adults with MASLD were included in the final analysis after application of the predefined eligibility criteria. Of these, 44 patients (20.2%) met the haemodynamic definition of CSPH, whereas 174 patients (79.8%) did not. The median HVPG in the overall cohort was 7.9 mmHg (IQR 6.21–9.55 mmHg). As expected, HVPG was higher in patients with CSPH than in those without CSPH, with median values of 11.56 mmHg (IQR 10.62–13.44 mmHg) and 7.42 mmHg (IQR 5.77–8.40 mmHg), respectively. This clear haemodynamic separation confirms the appropriateness of the outcome classification used in the present study.

### 3.2. Baseline Demographic and Clinical Characteristics According to CSPH Status

Baseline characteristics are summarised in Table 1. The median age of the cohort was 50.0 years (IQR 40.25–60.0 years), and age was comparable between patients without CSPH and those with CSPH, with medians of 50.5 years (IQR 41.25–60.0 years) and 49.0 years (IQR 38.25–60.0 years), respectively (*p* = 0.318). Body mass index was likewise similar between the two groups, with a median of 32.9 kg/m^2^ (IQR 29.72–35.55 kg/m^2^) among patients without CSPH and 32.7 kg/m^2^ (IQR 29.1–35.35 kg/m^2^) among those with CSPH (*p* = 0.589). Female sex accounted for 55.7% of patients without CSPH and 47.7% of those with CSPH, without a statistically significant between‐group difference (*p* = 0.398).

**Table 1:** Baseline demographic and clinical characteristics of the study population overall and according to clinically significant portal hypertension status.

Variable	Overall	No CSPH (*n* = 174)	CSPH (*n* = 44)	*p* value
Age (years)	50.0 (40.25–60.0)	50.5 (41.25–60.0)	49.0 (38.25–60.0)	0.318
BMI (kg/m^2^)	32.85 (29.7–35.55)	32.9 (29.72–35.55)	32.7 (29.1–35.35)	0.589
Female sex, *n* (%)	118 (54.1%)	97 (55.7%)	21 (47.7%)	0.398
Ascites present, *n* (%)	13 (6.0%)	5 (2.9%)	8 (18.2%)	0.001
Oesophageal/gastric varices present, *n* (%)	22 (10.1%)	9 (5.2%)	13 (29.5%)	< 0.001
Splenomegaly present, *n* (%)	66 (30.3%)	38 (21.8%)	28 (63.6%)	< 0.001

*Note:* Data are presented as median (interquartile range) for continuous variables and number (percentage) for categorical variables. *p* values were calculated using the Mann–Whitney *U* test for continuous variables and the Fisher exact test for categorical variables.

Abbreviations: BMI, body mass index; CSPH, clinically significant portal hypertension.

In contrast, clinical manifestations related to portal hypertension were substantially more frequent in the CSPH group. Ascites was present in eight of 44 patients (18.2%) with CSPH compared with five of 174 patients (2.9%) without CSPH (*p* = 0.001). Oesophageal or gastric varices were observed in 13 patients (29.5%) with CSPH and in nine patients (5.2%) without CSPH (*p* < 0.001). Splenomegaly was also more common among patients with CSPH, occurring in 28 patients (63.6%) compared with 38 patients (21.8%) in the no‐CSPH group (*p* < 0.001). Collectively, these findings indicate that although demographic characteristics were similar between groups, established clinical manifestations of portal hypertension were markedly enriched in patients with haemodynamically confirmed CSPH.

### 3.3. Comparison of Noninvasive Clinical, Laboratory and Ultrasound‐Derived Markers According to CSPH Status

**Figure 2:**
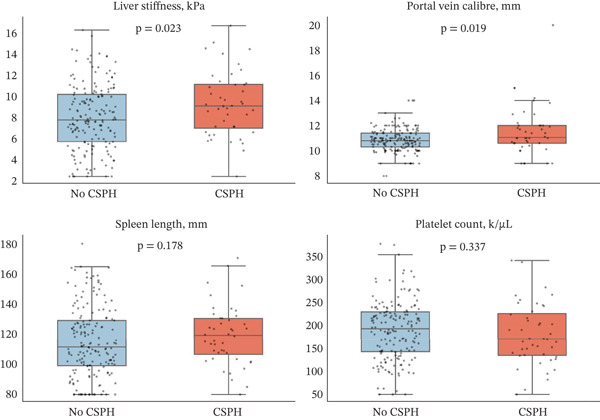
**Distribution of selected noninvasive markers according to clinically significant portal hypertension status**. Box‐and‐strip plots show the distribution of liver stiffness, portal vein calibre, spleen length and platelet count in patients with and without clinically significant portal hypertension. Liver stiffness and portal vein calibre were significantly higher in patients with CSPH, whereas spleen length was greater and platelet count was lower in the CSPH group without statistically significant between‐group differences.

The comparison of noninvasive markers according to CSPH status is presented in Table 2 and illustrated in Figure 2. Among the evaluated variables, liver stiffness and portal vein calibre were the markers that differed significantly between groups. Median liver stiffness was 9.15 kPa (IQR 7.04–11.19 kPa) in patients with CSPH compared with 7.82 kPa (IQR 5.77–10.23 kPa) in those without CSPH (*p* = 0.023). Portal vein calibre was likewise greater in the CSPH group, with a median of 11.05 mm (IQR 10.6–12.0 mm) compared with 10.8 mm (IQR 10.3–11.4 mm) in the no‐CSPH group (*p* = 0.019).

**Table 2:** Comparison of noninvasive clinical, laboratory and ultrasound‐derived markers between patients with and without clinically significant portal hypertension.

Variable	Overall	No CSPH	CSPH	*p* value
Liver stiffness (kPa)	8.14 (6.1–10.43)	7.82 (5.77–10.23)	9.15 (7.04–11.19)	0.023
Portal vein calibre (mm)	10.9 (10.32–11.5)	10.8 (10.3–11.4)	11.05 (10.6–12.0)	0.019
Spleen length (mm)	113.45 (100.21–129.74)	111.43 (99.08–129.04)	119.06 (106.51–130.38)	0.178
Platelet count (k/*μ*L)	189.35 (139.82–229.88)	193.38 (142.89–229.88)	170.41 (135.02–226.03)	0.337
Albumin (g/dL)	3.78 (3.41–4.18)	3.78 (3.41–4.17)	3.88 (3.53–4.24)	0.353
INR	1.16 (0.98–1.32)	1.17 (0.98–1.32)	1.12 (0.92–1.36)	0.688
AST (U/L)	53.56 (37.0–72.76)	54.04 (37.22–71.64)	46.98 (33.59–76.48)	0.72
ALT (U/L)	46.94 (29.88–64.38)	46.94 (30.08–63.73)	45.09 (29.83–70.07)	0.818
FIB‐4 score	2.19 (1.35–3.48)	2.19 (1.41–3.32)	1.81 (1.05–3.94)	0.625
APRI score	0.72 (0.49–1.06)	0.72 (0.49–1.03)	0.68 (0.41–1.29)	0.886

*Note:* Data are presented as median (interquartile range). *p* values were calculated using the Mann–Whitney *U* test.

Abbreviations: ALT, alanine aminotransferase; APRI, AST‐to‐platelet ratio index; AST, aspartate aminotransferase; CSPH, clinically significant portal hypertension; FIB‐4, fibrosis‐4 index; INR, international normalised ratio.

Other routinely available noninvasive parameters showed directional differences but did not achieve statistical significance. Spleen length was numerically greater in patients with CSPH than in those without CSPH, whilst platelet count tended to be lower in the CSPH group. However, neither difference reached the threshold for statistical significance. Similarly, albumin, INR, AST, ALT, FIB‐4 and APRI did not differ significantly between groups. These findings suggest that within this MASLD cohort, liver stiffness and portal vein calibre provided the clearest noninvasive signal associated with CSPH, whereas several conventional laboratory and morphometric markers showed more limited discriminatory value.

### 3.4. Logistic Regression Analysis of Factors Associated With CSPH

**Figure 3:**
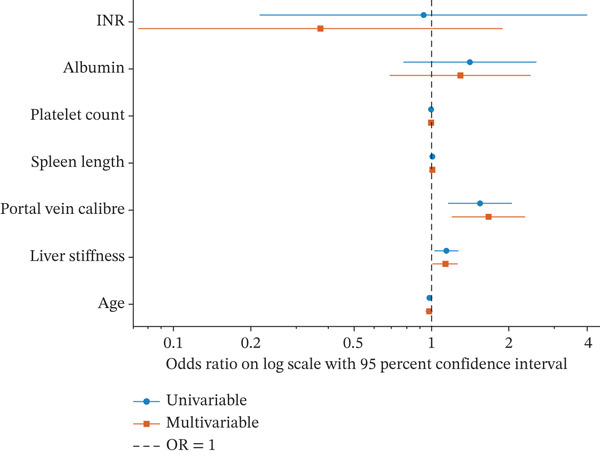
**Univariable and multivariable logistic regression analyses of factors associated with clinically significant portal hypertension**. Forest plot showing odds ratios and 95% confidence intervals for candidate variables associated with clinically significant portal hypertension in univariable and multivariable logistic regression models. Liver stiffness and portal vein calibre were significantly associated with CSPH in univariable analysis and remained independently associated in the multivariable model.

Univariable and multivariable logistic regression analyses are summarised in Table 3 and displayed in Figure 3. In univariable analysis, liver stiffness was significantly associated with CSPH, with an OR of 1.142 (95% CI 1.025–1.273; *p* = 0.016). Portal vein calibre was also significantly associated with CSPH, with an OR of 1.533 (95% CI 1.154–2.036; *p* = 0.003). By contrast, age, spleen length, platelet count, albumin and INR were not significantly associated with CSPH in the univariable models.

**Table 3:** Univariable and multivariable logistic regression analyses of factors associated with clinically significant portal hypertension.

Variable	Univariable OR	CI low	CI high	*p* value	Adjusted OR	Adjusted CI low	Adjusted CI high	Adjusted *p* value
Age	0.983	0.957	1.008	0.183	0.98	0.953	1.009	0.175
Liver stiffness (kPa)	1.142	1.025	1.273	0.016	1.134	1.014	1.269	0.028
Portal vein calibre (mm)	1.533	1.154	2.036	0.003	1.6	1.163	2.201	0.004
Spleen length (mm)	1.008	0.994	1.023	0.263	1.005	0.99	1.021	0.5
Platelet count (k/*μ*L)	0.998	0.992	1.003	0.371	0.997	0.991	1.002	0.246
Albumin (g/dL)	1.37	0.756	2.485	0.299	1.269	0.677	2.379	0.456
INR	0.958	0.222	4.136	0.954	0.564	0.113	2.807	0.484

*Note:* Results are presented as odds ratios with 95% confidence intervals and *p* values. Variables included in the multivariable model were age, liver stiffness, portal vein calibre, spleen length, platelet count, albumin and INR.

Abbreviations: CI, confidence interval; CSPH, clinically significant portal hypertension; INR, international normalised ratio; OR, odds ratio.

In the multivariable model, both liver stiffness and portal vein calibre remained independently associated with CSPH after adjustment for the other candidate variables. Liver stiffness showed an adjusted OR of 1.134 (95% CI 1.014–1.269; *p* = 0.028), whilst portal vein calibre showed an adjusted OR of 1.600 (95% CI 1.163–2.201; *p* = 0.004). The remaining variables did not retain independent statistical significance after adjustment. These results indicate that liver stiffness and portal vein calibre were the two noninvasive markers most consistently associated with CSPH in this cohort and that their associations were robust to multivariable adjustment.

### 3.5. Machine Learning Model Performance for the Prediction of CSPH

The predictive performance of the machine learning models is summarised in Table 4 and illustrated in Figure 4. Overall, model discrimination was modest across all evaluated approaches. Random forest achieved the highest cross‐validated AUROC at 0.632, followed by logistic regression at 0.604, whereas support vector machine showed the lowest discrimination at 0.514. Brier scores were similar across models, with values of 0.163 for random forest, 0.160 for logistic regression and 0.163 for support vector machine.

**Table 4:** Cross‐validated performance of machine learning models for the prediction of clinically significant portal hypertension.

Model	Cross‐validated AUROC	Brier score
Logistic regression	0.604	0.16
Random forest	0.632	0.163
Support vector machine	0.514	0.163

*Note:* Model performance was evaluated using five‐fold cross‐validation. Higher AUROC indicates better discrimination, whereas a lower Brier score indicates better overall prediction error.

Abbreviation: AUROC, area under the receiver operating characteristic curve.

Figure 4Discriminative performance and calibration of machine learning models for the prediction of clinically significant portal hypertension. (a) Receiver operating characteristic curves for the evaluated models using cross‐validated predictions from the study cohort. (b) Calibration plots comparing predicted probabilities with observed event rates across risk strata. Overall discriminative performance was modest, with random forest demonstrating the highest AUROC among the evaluated models.

**Figure:**
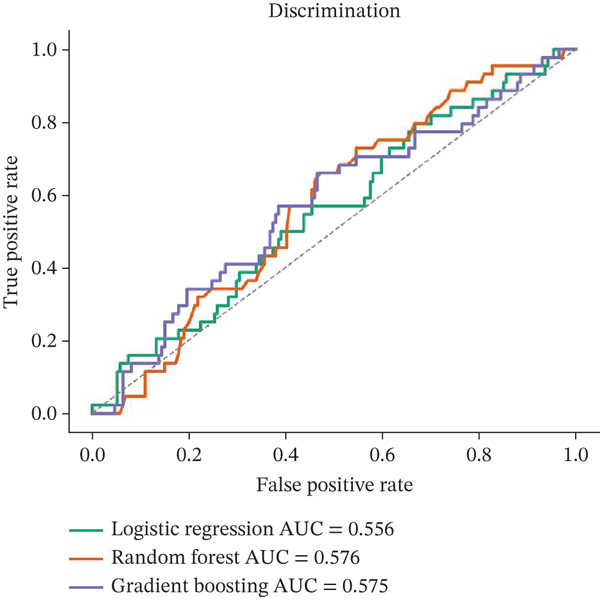
(a)

**Figure:**
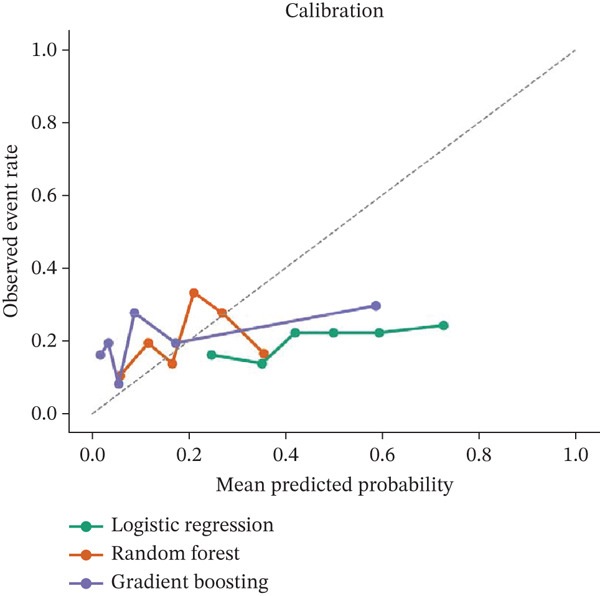
(b)

These findings indicate that although some noninvasive variables were associated with CSPH at the group level and in regression modelling, their combined predictive performance remained limited when applied within cross‐validated machine learning frameworks. Importantly, the results do not support overstatement of model utility. Rather, they suggest that routinely available clinical, laboratory and ultrasound‐derived variables may provide supportive information for preliminary risk stratification but are insufficient as stand‐alone tools for reliable identification of CSPH. In this regard, the machine learning results should be interpreted as exploratory and complementary to the regression analysis rather than as evidence of high diagnostic accuracy.

### 3.6. Integrated Summary of the Main Findings

Taken together, the results of the present study show that liver stiffness and portal vein calibre were the noninvasive markers most consistently associated with CSPH in this MASLD cohort. Both variables differed significantly between CSPH groups and remained independently associated with CSPH in multivariable logistic regression analysis. In contrast, other routinely available laboratory and morphometric variables, including platelet count, albumin, INR, spleen length, FIB‐4 and APRI, did not demonstrate independent associations in the adjusted model.

At the same time, the cross‐validated machine learning analyses demonstrated only modest predictive performance, indicating that the information contained in routinely available variables, although clinically relevant, was insufficient to support highly accurate stand‐alone prediction of CSPH. Therefore, the findings support the potential role of these variables as adjunctive tools for noninvasive risk stratification and selection of patients for further assessment rather than as substitutes for established diagnostic approaches such as HVPG measurement or dedicated elastographic evaluation, where available.

## 4. Discussion

In this retrospective cohort of adults with MASLD who underwent invasive haemodynamic assessment at a tertiary referral centre, CSPH was present in approximately one‐fifth of patients. This proportion is clinically meaningful because it identifies a substantial subgroup within a metabolically defined liver disease population who have already reached a haemodynamic threshold associated with portal hypertension‐related complications [14–17]. These findings reinforce that CSPH is an important issue in MASLD referral populations and merits explicit consideration during specialist evaluation.

The overall clinical and laboratory differences between patients with and without CSPH were modest. Platelet counts were lower in the CSPH group, and bilirubin levels were slightly higher, but these differences were not strongly discriminatory in isolation. Albumin and INR were broadly similar between groups, suggesting that conventional markers of hepatic synthetic dysfunction may remain relatively preserved even in patients with haemodynamically significant portal hypertension in this setting. This pattern is consistent with the natural history of MASLD, in which clinically relevant portal hypertension may develop before overt biochemical evidence of hepatic decompensation becomes apparent, particularly in selected referral cohorts. These findings highlight the limited value of routine laboratory parameters alone for identifying CSPH in this population [25, 29, 30].

By contrast, imaging‐related findings were more consistent with the expected pathophysiology of portal hypertension. Patients with CSPH had higher liver stiffness values and greater portal vein calibre, whilst spleen dimensions also tended to be larger, although not all morphometric differences reached statistical significance on univariable comparison. These findings are biologically coherent and align with the concept of the liver‐spleen axis, in which increasing intrahepatic vascular resistance is accompanied by portal venous remodelling, splenic congestion and platelet sequestration [22, 31, 32]. Although much of the previous literature has focused on spleen stiffness, the present study is more directly applicable to routine practice because it examines simple ultrasound morphometrics and standard laboratory variables that are widely available in general hepatology care [27, 33–36].

The regression analyses further clarify the relative contribution of these variables. In the multivariable model, portal vein calibre and liver stiffness emerged as the most informative independent predictors of CSPH, whereas other variables lost statistical significance after adjustment. This result is plausible because portal vein calibre may reflect chronic haemodynamic adaptation within the portal circulation, whilst liver stiffness captures the structural substrate that underlies increased intrahepatic resistance. Spleen size and platelet count, although clinically relevant, are likely influenced by a wider range of biological and technical factors and may therefore contribute less consistently when considered alongside stronger direct markers of portal haemodynamic and fibrosis burden. Taken together, these findings suggest that the most useful routine predictors of CSPH in MASLD are those that most directly reflect the haemodynamic and structural consequences of portal hypertension [28].

The machine learning analyses yielded only modest discrimination, with the random forest model performing best, followed by the support vector machine, whereas logistic regression achieved slightly lower discrimination [24, 26]. However, the differences between approaches were not large, and none of the models demonstrated sufficient accuracy to justify stand‐alone clinical use. This is an important negative finding. It suggests that when only routine ultrasound, elastography, anthropometric and laboratory variables are available, more flexible algorithms do not necessarily overcome the limited signal contained within the input data. In this setting, the constraint appears to be less the modelling technique itself than the underlying informativeness of the routinely available variables.

These findings should also be interpreted within the broader biological context of MASLD. MASLD is increasingly recognised as a systemic cardiometabolic disorder with a close and bidirectional relationship with cardiovascular disease, rather than an isolated liver condition. Shared mechanisms such as insulin resistance, visceral adiposity, adipocyte dysfunction, oxidative stress, chronic low‐grade inflammation, endothelial dysfunction and fibrosis‐related pathways contribute to both hepatic injury and cardiovascular remodelling [10–13]. Through these intersecting mechanisms, MASLD has been linked to coronary artery disease, atrial fibrillation, heart failure and increased mortality, whilst coexisting cardiometabolic disease may also modify hepatic phenotype and accelerate the progression of liver disease [11–13]. This broader systemic framework is relevant to the present findings because it offers a plausible explanation for why routine liver‐focused and ultrasound‐based variables provide only moderate discrimination: The phenotype of MASLD‐related CSPH is shaped by heterogeneous, multisystem processes that are not fully captured by standard hepatic measurements alone.

From a clinical perspective, the present results suggest that routinely available ultrasound and laboratory markers still have value, but mainly as components of a structured risk assessment strategy rather than as definitive diagnostic tools. Portal vein calibre and liver stiffness appear to provide the strongest noninvasive signal, whilst spleen morphometrics and platelet count may offer supportive contextual information. This pattern suggests that these variables retain clinical value as part of an overall assessment framework, particularly when interpreted alongside the broader metabolic profile and conventional indicators of liver disease severity. Their role may be less as definitive stand‐alone predictors and more as accessible markers that help identify patients in whom the pretest probability of CSPH is higher.

More broadly, these findings fit with the current movement in MASLD care toward structured noninvasive risk stratification. As the number of patients with steatotic liver disease continues to rise, clinicians increasingly need practical ways to distinguish those who may have clinically important portal hypertension from those at lower risk. Within that framework, routinely collected ultrasound and laboratory features remain attractive because they are inexpensive, familiar and scalable. The present data suggest that such variables can contribute meaningful information but also demonstrate that their performance is inherently moderate when used without more specialised markers. This nuance is important and should inform how such models are interpreted in both research and clinical settings.

Overall, the findings from this study do not support the use of any single routine marker as a reliable identifier of CSPH in MASLD. Rather, they suggest that several routinely available features, including portal vein calibre, liver stiffness, spleen morphometrics and platelet count, reflect the haemodynamic and structural changes associated with portal hypertension and may be most informative when considered together. The modest predictive performance across all models underscores the difficulty of accurate noninvasive CSPH prediction using routine data alone. In that sense, this study contributes a realistic MASLD‐specific perspective to the noninvasive assessment literature and helps define both the promise and the present boundaries of routine data–based prediction.

## 5. Conclusion

In this retrospective cohort of adults with MASLD undergoing invasive haemodynamic assessment, routinely available ultrasound, elastography and laboratory variables showed only modest ability to distinguish patients with CSPH. Variables reflecting portal haemodynamics, splenic changes and platelet‐related effects appeared to provide the most informative noninvasive signal, whereas routine biochemical markers alone offered limited discrimination. These findings suggest that standard clinical data capture some of the structural and haemodynamic consequences of portal hypertension but remain insufficient for accurate stand‐alone noninvasive identification of CSPH.

Predictive performance was modest across both conventional regression and machine learning approaches, with no clear evidence that more complex algorithms conferred a substantial advantage over simpler models. The results therefore support a cautious interpretation of routine noninvasive data in MASLD. Such variables may still be useful for preliminary risk stratification and for identifying patients who may benefit from closer assessment, but they should be regarded as adjunctive rather than definitive tools for CSPH detection [22, 23, 37].

Overall, this study adds MASLD‐specific evidence to the literature on noninvasive portal hypertension assessment by defining both the potential value and the current limitations of routine data–based prediction. These findings support continued efforts to refine simple, interpretable risk models whilst underscoring the need for external validation and prospective multicentre evaluation before such approaches can be incorporated into clinical decision pathways.

## 6. Limitations

Several limitations should be considered when interpreting these findings. First, this was a retrospective study conducted at a single tertiary referral centre, and the requirement for invasive haemodynamic assessment, together with available routine noninvasive data, resulted in a clinically selected cohort. This may limit generalisability and introduce referral and selection bias, particularly because the study population is likely to have had a higher prevalence of advanced liver disease and portal hypertension than broader MASLD populations.

Second, several predictors were derived from routine clinical ultrasound rather than a fully standardised research imaging protocol. Although examinations were subject to predefined quality criteria, measurements such as spleen dimensions and portal vein calibre remain operator‐dependent and may be influenced by image quality and technical variation, potentially attenuating discrimination between groups and reducing model performance.

Third, the sample size and number of CSPH events were modest relative to the number of predictors evaluated. This may have limited statistical power, reduced the precision of effect estimates and affected the stability of model performance. Although internal cross‐validation was used to reduce optimism, external validation in independent MASLD cohorts is still required before these findings can be generalised.

Fourth, the analysis was cross‐sectional and focused on CSPH status at a single time point rather than longitudinal clinical outcomes. The findings should therefore be interpreted as relating to the noninvasive identification of haemodynamic status rather than the prediction of future liver‐related events.

**Nomenclature**
ALT: alanine aminotransferase
APRI: aspartate aminotransferase‐to‐platelet ratio index
AST: aspartate aminotransferase
AUROC: area under the receiver operating characteristic curve
BMI: body mass index
CI: confidence interval
CSPH: clinically significant portal hypertension
EGD: eosophagogastroduodenoscopy
HVPG: hepatic venous pressure gradient
INR: international normalised ratio
LR: logistic regression
MASLD: metabolic dysfunction–associated steatotic liver disease
MELD: Model for End‐Stage Liver Disease
ML: machine learning
NAFLD: nonalcoholic fatty liver disease
NITs: noninvasive tests
OR: odds ratio
PPV: positive predictive value
NPV: negative predictive value
RF: random forest
ROC: receiver operating characteristic
SD: standard deviation
SVM: support vector machine
US: ultrasound

## Author Contributions

Conceptualization: N.Y.S. and C.S.P. Methodology: N.Y.S. and T.A.T. Software: N.Y.S. Validation: N.Y.S., L.M.N. and B.E.D. Formal analysis: N.Y.S. Investigation: N.Y.S. and T.A.T. Resources: C.S.P. and B.E.D. Data curation: N.Y.S. and L.M.N. Writing—original draft preparation: N.Y.S. Writing—review and editing: N.Y.S., T.A.T., L.M.N. and B.E.D. Visualization: N.Y.S. and T.A.T. Supervision: C.S.P. Project administration: C.S.P. and T.A.T. Funding acquisition: C.S.P.

## Funding

No funding was received for this manuscript.

## Ethics Statement

This study was conducted in accordance with the Declaration of Helsinki and applicable institutional requirements for retrospective research using clinical data. The study protocol was reviewed and approved by the Local Ethics Committee of I.M. Sechenov First Moscow State Medical University, Ministry of Health of the Russian Federation (Sechenov University) (extract from Protocol No. 27‐24; approval date 07 November 2024). HVPG procedures and associated clinical assessments were performed as part of routine clinical care or approved research protocols, as applicable. All data were deidentified before analysis in accordance with local regulatory and ethical requirements. As this was a retrospective analysis of deidentified records with no direct patient contact or study‐specific intervention, the requirement for informed consent was waived by the ethics committee.

## Consent

Patient consent was waived due to the retrospective design and exclusive use of deidentified data extracted from existing electronic health records and archived imaging, with no direct interaction or intervention.

## Conflicts of Interest

The authors declare no conflicts of interest.

## Data Availability

Deidentified data underlying this study are available from the corresponding author upon reasonable request and in accordance with institutional and data protection policies. Aggregated results and analysis code (where applicable) can be shared to support reproducibility whilst preserving participant privacy.
